# Feasibility and acceptance of exercise recommendations (10,000 steps a day) within routine German health check (Check-Up 35/GOÄ29)—study protocol

**DOI:** 10.1186/s40814-016-0092-9

**Published:** 2016-09-07

**Authors:** Christine Graf, Stefanie Schlepper, Carina Bauer, Nina Ferrari, Stefan Frank, Lena Gartner, Svenja Gehring, Rudolf Henke, Walter Lehmacher, Hans-Michael Steffen, Sabine Schindler-Marlow, Katharina Sternal

**Affiliations:** 1 Institute of Movement and Neuroscience, German Sport University, Am Sportpark Müngersdorf 6, 50933 Cologne, Germany; 2 Northrhine Medical Association, 40474 Düsseldorf, Germany; 3 Institute of Medical Statistics, Informatics and Epidemiology, University of Cologne, 50924 Cologne, Germany; 4 Clinic for Gastroenterology and Hepatology, University Hospital of Cologne, 50937 Cologne, Germany

**Keywords:** Motivational interviewing, Feasibility, Brief intervention, Pedometers, General practitioners, Overweight

## Abstract

**Background:**

Benefits of exercise to prevent non-communicable diseases are well-documented. Limited data exists to promote physical activity in healthy but sedentary and/or overweight people. Brief interventions within routine German health checks may be an effective way to reach these patients.

**Methods/design:**

The quasi-experimental, multi-center prospective feasibility study is designed for general practices in Cologne (intervention group) and Düsseldorf (control group), up to 20 per region. Eight to 10 inactive and/or overweight patients per practice will be recruited for a total of 300. General practitioners and at least one of their nurses for the intervention group will be trained in motivational interviewing and familiarized with low-threshold recommendations for exercise (activities of daily life (ADL), target of 10,000 steps/day) and additional tools (pedometers, activity diaries). Participants in the control group will only receive general advice (150 min of exercise/week). The primary aims are to evaluate the feasibility of this intervention and to determine whether it is possible to reach a mean increase of 1000 steps/day in the target group within 6 months. Secondary objectives focus on the number of patients who reach a target of 10,000 steps/day and their improvements in quality of life and decrease in body mass index, waist circumference, and blood pressure.

**Discussion:**

The study will assess whether it is feasible to run brief interventions within the GP setting can promote an active lifestyle in overweight and/or inactive patients.

**Electronic supplementary material:**

The online version of this article (doi:10.1186/s40814-016-0092-9) contains supplementary material, which is available to authorized users.

## Background

Modern lifestyle is characterized by low physical activity levels and sedentary behavior during work, daily life, and leisure time [1]. According to current German health surveys, 51.7 % of men and 49.5 % of women exercise regularly for at least 1 h/week, but only 25.4 % of men and 15.5 % of women reach the 150 min/week that are recommended by the World Health Organization [2]. One possible consequence associated with a sedentary lifestyle is the growing number of overweight and obese people and increasing incidences of cardio-metabolic diseases [3, 4]. Current figures show that 67 % of German men and 53 % of German women are overweight (≥25 kg/m^2^) or obese (≥30 kg/m^2^) [5]. Therefore, effective preventive strategies in terms of lifestyle changes and an increase of physical activity are warranted. A recent meta-analysis revealed that moderate physical exercise led to a reduction in cardiovascular events of approx. 20 to 30 % in men and approx. 10 to 20 % in women [6]. Prescribed as a corresponding medication, physical exercise has also been shown to have similar effects on stroke, coronary heart disease (CHD), heart failure, and prediabetes [7]. An increase of 1000 steps in the daily number of steps may reduce the risk of metabolic syndrome by 10 % [8] and the achievement of more than 10,000 steps per day can lead to a reduction of the body mass index (BMI) and arterial blood pressure [9–11]. However, this goal is usually achieved by only about 15 % of study participants [12, 13]. General practitioners (GPs) may play a significant role in health counseling, patient’s motivation, and advising patients about how to change their lifestyles [14]. Supporting counseling of inactive patients with additional aids, e.g., prescribing more physical activity, has led to a considerable increase in physical activity levels [15]. Therefore, the Ärztekammer Nordrhein (Northrhine Medical Association) and the Deutsche Sporthochschule Köln (German Sport University Cologne) developed the “10,000 step study”, consisting of a brief intervention with motivational interviewing (MI) and the use of additional tools such as pedometers and activity diaries. Pedometers constitute an easy-to-use and low-cost method of promoting self-motivation in patients by enabling them to monitor and integrate (more) physical exercise, i.e., walking, into their daily living. The key message of achieving a “target of 10,000 steps a day” turned out to be the most important predictor of success [9–11]. This study will target inactive and/or overweight adults (BMI ≥25 kg/m^2^) visiting their GPs for routine health checks (e.g., Check-Up 35/GOÄ29; Gebührenordnung für Ärzte; medical fee regulation). Utilizing motivational interviewing, practitioners are able to convey the crucial messages regarding an active lifestyle (i.e., the 10,000 steps/day recommendation) despite the generally short patient-practitioner interaction [16–18].

Two main outcomes at the GP and patient level are targeted, respectively. The primary goal at the GP level is to evaluate the feasibility of this brief intervention within the setting of routine health checks. At the patient level, the main feasibility outcome will be to see if a mean increase of 1000 steps/day can be achieved within a period of 6 months. Secondary objectives focus on the number of patients reaching a target of 10,000 steps/day, improvements of patients’ quality of life (SF 36), and decrease of BMI, waist circumference, and systolic and diastolic blood pressure to determine whether these are appropriate short-term measurements for monitoring behavior change.

## Methods/design

### Design

The 10,000 step study is a quasi-experimental, multi-centric prospective feasibility study. A quasi-experimental design is chosen because it is not possible to randomize the intervention according to individual patient or practitioners within the services at this moment. Ethical approval for the study was obtained from the ethics commission of the German Sport University Cologne (No. 18/2013). This protocol complies with the SPIRIT guideline of writing protocols.

### Target group and sample size

The target group will consist of overweight (BMI ≥25 kg/m^2^) [19] and/or inactive patients (<1 h exercise/week) visiting GPs for routine health checks (Check-Up 35 (age ≥35 years) and/or GOÄ29 (age ≥18 years)).

The primary outcome is the increase in steps/day after 6 months. In the intervention group (IG), an additional mean increase of 1000 steps/day in contrast to the controls is a feasible difference with a risk reduction in metabolic syndrome, BMI, fat mass, etc. [8]. It is expected that in the treatment group there is a larger mean increase of 1000 steps/day with a standard deviation of 2200 steps as a conservative estimation. Then, a sample size of 105 patients per group would be required in order to detect a difference between groups at a significance level of 0.05 (two-tailed) with a power of 0.90. Considering an attrition rate of approximately 30 % over the course of the study, a total of 300 subjects (150 for each group) will be enrolled. The intra-cluster correlation (ICC) is expected to be small but is unknown, and consequently, the power of this study may be reduced. Success rates like the rate of patients reaching an increase of more than 1000 steps or reaching the target of 10,000 can be estimated with 95 % confidence intervals reduced than ±10 %.

### Recruitment

A total of 714 GPs in Cologne will be informed by the Northrhine Medical Association and invited to take part in the study. The first 20 practices will be recruited into the study and trained to provide brief interventions. Likewise, a control group (CG) will be selected at a different location (Düsseldorf; in total 426 GPs) to prevent regional overlaps, mixing of study participants and participating physicians talking to each other. Düsseldorf has been selected because of its similarity and comparability in terms of size, region, and practitioners’ clientele.

**Fig. 1:**
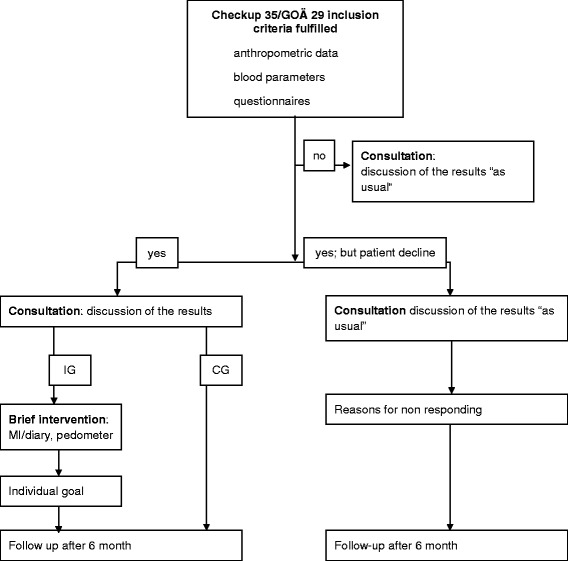
Flow diagram of GPs through the study. Procedure of Cologne GPs (IG) and Düsseldorf GPs (CG)

Data for all patients registered for health checks (Check-Up 35/GOÄ29; anthropometric data, BMI, waist circumference, and blood pressure) will be documented. Patients who meet the inclusion criteria will be invited to participate in the study until the planned number of 8 to 10 participants per GP is achieved. Then, anthropometric data, arterial blood pressure (after 5 min of sitting) and questionnaires regarding sociodemographic factors including personal and family history will be collected at baseline (T1). Furthermore, individual lifestyle factors and SF-36 [20] health surveys will be collected both at baseline (T1) and as a follow-up 6 months later (T2). Within the intervention group, the brief intervention will be performed. The controls receive “usual care” only, i.e., general information given verbally about the goal of exercising 150 min/week. In case of non-participation, both the patients’ data and their reasons for non-participation will be documented providing their consent in written form (Fig. 1).

### Brief intervention in the intervention group

The brief intervention targets overweight and/or inactive patients with the intention to increase their daily steps and therefore physical activity in daily life. The intervention group’s GPs and at least one of their nurses will be informed about current exercise recommendations (focusing on ADL, 10,000 steps/day, and 150 min of exercise/week). Patients will be issued pedometers with instructions about their use and exercise diaries to document their physical activities (type; duration in minutes, steps/day, and mental disposition at the time (positive, neutral, negative)). In addition, they will be taught how to conduct motivational interviewing to communicate the “10,000 steps a day” message during every contact with the participating patients. This training will be delivered through a 3-h MI workshop conducted by an experienced coach. The participants will learn the basics and fundamental principles of MI as well as the use of specific skills, e.g., empowerment, ambivalence, the decision balance schedule, and reflective listening [17]:

Principle 1—Express empathy. Put yourself in the patient’s shoes so that you can understand his or her point of view. A basic empathetic attitude represents the foundation for motivational interviewing that is characterized by appreciative and sympathetic understanding.

Principle 2—Develop discrepancies. One of the intervention’s goals will be to make the patient aware that their current behaviors (e.g., sedentary lifestyle) are impeding them from achieving their goals (e.g., healthy life) and to convey an understanding of benefits and disadvantages between the old and new lifestyle.

Principle 3—Roll with the resistance instead of opposing it. The motivational interviews will not focus on persuading patients to change their behaviors against their own resistance but instead will be more concerned with accentuating the patients’ own responsibility for their individual actions.

Principle 4—Support self-efficacy. The success of patients’ actions will be greatly facilitated if they are supported in feeling optimistic and effective concerning their ability to change.

These principles have been specifically developed for this study and for the physician and nurses to patient communication during health checks as well as the ongoing exchanges with medical staff (by phone and at the practices) on the basis of the adopted Transtheoretical Model of Prochaska and DiClemente [18] (see Table 1). In addition, all techniques will be practiced using interactive class activities, role plays, and simulated patient feedback. Subsequent supervisions are provided as an option within the first 4 weeks after the workshop.

**Table 1:** Representation of the various stages of change in the behavior of patients

Stage (modified TTM)	Patient	GP	Principle (modified MI)
Non-intent (pre-contemplation)	Does not feel the need to change and/or has not (yet) thought about possible change	Provision of information about the “10,000 step study” with the aim of encouraging a willingness to change	1
The creation of intent (contemplation)	Is undecided and ambivalent towards change	Talking about the “pros” (greater quality of life through exercise) and “cons” (not enough time) of old and new behaviors; dealing flexibly with resistance; strengthening of self-efficacy	2; 3; 4
Preparation	Is increasingly willing to change; plans are made	Encouraging a feeling of optimism about the ability to exercise in day-to-day life	4
Action	Acts!	Encouragement (by viewing the exercise diaries) and appreciation of changes	1; 4
Maintenance	“Saves” the new behavior	Encouragement and appreciation of changes by medical staff	1; 4
Termination	Always behaves in the new way	Encouragement and appreciation of changes within the framework of the final checkup	1; 4

The various stages of change in the behavior of patients and their accompaniment by GPs and participating medical staff during the study based on the use of the motivational interview (MI) [24] and the Transtheoretical Model (TTM) developed by Prochaska and DiClemente [18]

This kind of brief intervention will be carried out within the scope of Check-Up 35/GOÄ29. GPs may utilize the situation and any results from the checkup to discuss healthy living with the patient. This study is structured in a way that enables GPs to conduct MI with their patients and encourage them to take action themselves. Therefore, patient participation in the intervention is a key factor. To facilitate patient understanding, the “10,000 steps a day” message will be communicated and underscored by issuing pedometers and exercise diaries. The study will also require medical staff to ask patients to attend the practice once a month so that they may accompany the maintenance phase by jointly reviewing the exercise diaries with patients and make positive and appreciative comments about their efforts (Additional file 1). This sharing of the tasks takes into account the time, finances, and staffing constraints that GPs currently face. At the final consultation (after 6 months), GPs will be able to remark positively on the patient’s changes in behavior and thus contribute to the continuation of the new behaviors. Patients may then be asked throughout all routine health checkups about their progress or relapse they may have experienced (relapse is always possible when people try to change lifestyles). Relapse management should, therefore, be integrated into the “motivational interviewing,” which will also incorporate an empathetic way of dealing with “failures” and of creating new targets based on the phases described in the table.

### Control group

The GPs and medical staff in the control group will also be trained. The focus, however, will be placed on communicating the message that it is necessary to exercise for 150 min a week. In addition to this verbally given recommendation, local sports clubs may or may not be suggested. Specific communication methods and the structured manner of proceeding during the brief intervention will not be part of this training. Pedometers will only be handed out to monitor (day-to-day) activities for 14 days at the beginning and the end of the study period.

### Patient data

Weight and height, BMI, waist circumference, and arterial blood pressure as well as calculated BMI will be determined at baseline (T1) and at the follow-up after 6 months (T2). Body weight will be measured using the standard methods with the patient wearing light clothes and no shoes; the systolic and diastolic blood pressures will be measured after the patients have been sitting calmly without exertion for 5 min. BMI will be calculated and classified using the World Health Organization’s specifications (BMI = body weight/body size^2^) [19]. Patients with values ≥25 kg/m^2^ should be deemed overweight, and those with values ≥30 kg/m^2^ should be regarded as obese.

At both consultations, patients will be given a modified IPAQ ((International Physical Activity Questionnaire, 2002) Additional file 2) for documenting their individual lifestyles. All activities will be documented in exercise diaries, which will be used to determine the amount and intensity of active minutes per week at baseline and at the follow-up. The number of steps is collected for the whole period in the intervention group. In the control group, the participants will take the pedometer only for the 2 weeks at the beginning and the end of the study period. Only the steps per week from the baseline and follow-up data collection will be factored into the analysis. The follow-up model HJ-322U of the valid, accurate, and reliable HJ-720IT Walking style pro 2.0 (Omron, Kyoto, Japan) will be utilized in the present study to measure free-living step counts [21].

The general state of health and/or the health-related quality of life and the effects of the lifestyle intervention will be investigated with SF 36 [20] that will establish the aspects of physical functioning capacity, physical role functioning, bodily pain, general condition of health, vitality, social functioning capacity, emotional role functioning, and mental well-being.

### Feasibility outcomes

The feasibility will be tested with guide-assisted interviews with each GP and the medical staff and the use of focus group discussions at the end of recruitment in both regions. Additionally, all GPs and their nurses will be subjected individually to a guided interview on the phone. Questions will address recruitment rate, willingness of the patients to participate, reasons for participating or not participating, adherence, and attrition due to the intervention or specific patients’ characteristics (e.g., gender, age) to assist in a sample size for a larger-scale cluster-randomized trial. They will be asked whether it is believed that the practice and patients are suitable and whether the execution of the brief intervention (in the IG) and the recommendations (to the CG) is possible. The questions will be both open and closed, whereby the 5-tier Likert scale (strongly agree to strongly disagree) will be utilized in designing the latter. The open questions should represent additional points of view, which can be clustered thematically afterwards. Additionally, one to two focus group discussions with the GPs are planned in each city (max. 10 per discussion). The central questions for the groups will be deducted from the phone interview responses to allow further feedback regarding approaches, patient acceptance, implementation in practices, and the supporting role of the MFAs and their possible implementation.

### Data analysis

Usual descriptive statistics will be used to summarize the results. Means per group, mean differences between the groups, rates, and rate differences will be analyzed with 95 % confidence intervals. Multivariate analyses with linear and logistic regressions will be performed in order to explore the practice effect and further potential covariates and cofactors. The design effect of clustering (and intra-cluster correlation) will be calculated to aid sample size estimation for a future cluster-randomized trial.

## Discussion

This study intends to examine the feasibility of a brief intervention; especially motivational interviewing at GP practices carried out within the scope of routine German health checks. It aims to analyze effects on patients’ physical activity, anthropometric data (weight, BMI, waist circumference), arterial blood pressure, activity level, and health-related quality of life. In addition, sociodemographic factors of the patients and GPs are taken into consideration due to their essential impact on individual lifestyle [22, 23]. People with a lower level of education pose a challenge because medical counseling and explanations require more expenditure of time.

The authors believe that this type of brief intervention may not only be employed within the framework of checkups in order to successfully trigger lifestyle changes but that it also has the potential to generally improve the communication between physicians and patients. This is due to its patient-oriented motivating counseling that takes the social situation and individual preferences regarding lifestyle modification into consideration. Because the nurses are deliberately integrated into the setup of the study, it can be anticipated that the cooperation between GPs and their nurses in the supervision of patients over the 6-month study period may have a positive effect on the practice setting. The increased responsibility amongst the staff may potentially strengthen and enhance their position in the practice and thus lead to an improved working atmosphere.

The results may strengthen the role of GPs, including their medical staff, in the prevention of non-communicable diseases and health promotion. Additionally, the study will probably show how exercise recommendations embedded within the framework of routine health checks (Check-Up 35/GOÄ29) can be implemented effectively and efficiently as preventative means for non-communicable diseases. The results of this study will enable us to gather information on the feasibility and the implementation in routine practice. The estimated effect sizes and cluster design effect with the explored prognostic factors will enable the planning of a future cluster-randomized trial.

## Additional files

Additional file 1: Exercise diary. (PDF 1341 kb)
Additional file 2: Modified IPAQ (International Physical Activity Questionnaire, 2002). (PDF 1377 kb)

## Abbreviations

ADL: Activities of daily life
BMI: Body mass index
CG: Control group
CHD: Coronary heart disease
GPs: General practitioners
IG: Intervention group
IPAQ: International Physical Activity Questionnaire
MI: Motivational interviewing
