# Supplementary material for: Feasibility and acceptance of exercise recommendations (10,000 steps a day) within routine German health check (Check-Up 35/GOÄ29)—study protocol
Source: Pilot Feasibility Stud. 2016 Sep 7;2:52. doi: 10.1186/s40814-016-0092-9 (PMC5154129; doi:10.1186/s40814-016-0092-9)
Supplement: Additional file 1: — Exercise diary. (PDF 1341 kb) [file 40814_2016_92_MOESM1_ESM.pdf]

Ärztekammer Nordrhein

Deutsche Sporthochschule Köln
German Sport University Cologne

Mein Bewegungs-Tagebuch

10.000 Schritte für Ihre Gesundheit

Woche:

vom: **bis:**

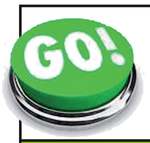

	**Meine sportliche Aktivität** Art der Bewegung (Fahrrad, Schwimmen, Tennis, Kegeln etc.)	**Dauer** (in Min.)	**Meine Schritte** (Schrittzähler-Schritte/ Tag)	**Meine Stimmung** (Tag)
Montag				
Dienstag				
Mittwoch				
Donnerstag				
Freitag				
Samstag				
Sonntag				

Anleitung zum Ausfüllen:
Notieren Sie **an jedem Wochentag** die **Art ihrer körperlichen Aktivität** und die **Dauer** direkt daneben. Lesen Sie am Ende des Tages die **Zahl Ihrer Schritte** auf Ihrem Schrittzähler ab und tragen den Wert in der Spalte **Meine Schritte** ein. Bewerten Sie in der Spalte **Meine Stimmung**, wie Sie sich am Tag gefühlt haben und kreuzen den entsprechenden Smiley an.
