# Supplementary material for: Feasibility and acceptance of exercise recommendations (10,000 steps a day) within routine German health check (Check-Up 35/GOÄ29)—study protocol
Source: Pilot Feasibility Stud. 2016 Sep 7;2:52. doi: 10.1186/s40814-016-0092-9 (PMC5154129; doi:10.1186/s40814-016-0092-9)
Supplement: Additional file 2: — Modified IPAQ (International Physical Activity Questionnaire, 2002). (PDF 1377 kb) [file 40814_2016_92_MOESM2_ESM.pdf]

Deutsche Sporthochschule Köln
German Sport University Cologne

Basisfragebogen

Alle Patientendaten unterliegen der ärztlichen Schweigepflicht und werden nur in anonymisierter Form weitergeleitet.

10.000 Schritte für Ihre Gesundheit

Datum: / /

© Deutsche Sporthochschule Köln, Ärztekammer Nordrhein

Allgemeiner Hintergrund

Ihr Geburtsdatum: ____________________

Ihr Geschlecht: ❏ weiblich ❏ männlich

1. Ihr Familienstand?

❏ ledig ❏ verheiratet ❏ nicht eheliche Lebensgemeinschaft
❏ verwitwet ❏ geschieden / getrennt lebend

2. Wie ist Ihr höchster schulischer Abschluss?

❏ Volksschul-/Hauptschulabschluss ❏ Mittlere Reife, Realschulabschluss
❏ Fachhochschulreife ❏ Abitur
❏ Anderer Schulabschluss: ____,

wie viele Schulklassen: (Anzahl)

❏ Nichts davon, ich habe (noch) **keinen** Schulabschluss

3. Sind Sie aktuell berufstätig?

❏ Voll berufstätig ❏ In Teilzeit berufstätig ❏ Nicht erwerbstätig

4. Welche Sprache wird bei Ihnen zu Hause hauptsächlich gesprochen? ____________________

I Und nun zu Ihren Aktivitäten in Alltag und Beruf

5. Wie gestaltet sich Ihre berufliche Tätigkeit hauptsächlich?

❏ Ausschließlich sitzende Tätigkeiten (z. B. Bürotätigkeit, ...)

❏ Überwiegend sitzende Tätigkeiten (z. B. Kraftfahrer, Laborant, ...)

❏ Mäßige Bewegung (z. B. Hausfrau, Verkäufer, ...)

❏ Intensive Bewegung (z. B. Fensterputzer, Postzusteller, ...)

6. An wie vielen Tagen pro Woche gehen Sie aktuell in Ihrem Alltag zu Fuß oder fahren mit dem Rad, um von einem Ort zum anderen zu gelangen (inkl. Ihres Arbeitsweges)?
Bitte geben Sie auch die durchschnittliche Minutenzahl an.

______ Tage pro Woche, für jeweils etwa _______ Minuten

7. An wie vielen Tagen pro Woche verrichten Sie aktuell körperliche Aktivitäten wie das Tragen von Lasten oder Arbeiten im Haus, Hof oder im Garten und ähnliches?
Bitte geben Sie auch die durchschnittliche Minutenzahl an.

______ Tage pro Woche, für jeweils etwa _______ Minuten

II Zu Bewegung/Sport in der Freizeit – bitte berücksichtigen Sie nicht Ihre Angaben zu Bewegung in Alltag und Beruf

8. Sind Sie momentan Mitglied in einem Sportverein? ❑ nein ❑ ja

9. Welche Sportart(en) betreiben Sie aktuell regelmäßig, d.h. mindestens ein Mal pro Woche?
Wie oft pro Woche für **wie viele Minuten pro Mal**?

Sportart: ____________________ ☐ mal pro Woche _______ Minuten pro Mal

Sportart: ____________________ ☐ mal pro Woche _______ Minuten pro Mal

Sportart: ____________________ ☐ mal pro Woche _______ Minuten pro Mal

10. Welche Sportart betreiben Sie zusätzlich, d.h. weniger als zwei- bis dreimal im Monat?
Wie oft pro Monat für **wie viele Minuten pro Mal**?

Sportart: ____________________ ☐ mal pro Monat _______ Minuten pro Mal

Sportart: ____________________ ☐ mal pro Monat _______ Minuten pro Mal

Sportart: ____________________ ☐ mal pro Monat _______ Minuten pro Mal

11. Fühlen Sie sich in Ihren körperlichen Funktionen eingeschränkt? ❑ ja ❑ nein

Wenn ja, inwiefern: ____

____

____

III Sitzende Tätigkeiten, z.B. Lesen, Fernsehen, PC, im Alltag, Beruf und Freizeit

12. Wie viele Stunden verbringen Sie durchschnittlich mit sitzender Tätigkeit an einem Wochentag (Mo–Fr)? Ergänzen Sie falls nötig die Tabelle.

beruflich			privat		
Bildschirmarbeit		Std.	PC		Std.
Büroarbeit		Std.	TV		Std.
Auto		Std.	Lesen/Schreiben		Std.
Kasse		Std.	Autofahren		Std.
______		Std.	______		Std.
______		Std.	______		Std.
______		Std.	______		Std.
______		Std.	______		Std.

13. Wie viele Stunden verbringen Sie durchschnittlich mit sitzender Tätigkeit an einem Wochenendtag (Sa/So)? Ergänzen Sie falls nötig die Tabelle.

beruflich			privat		
Bildschirmarbeit		Std.	PC		Std.
Büroarbeit		Std.	TV		Std.
Auto		Std.	Lesen/Schreiben		Std.
Kasse		Std.	Autofahren		Std.
______		Std.	______		Std.
______		Std.	______		Std.
______		Std.	______		Std.
______		Std.	______		Std.

IV Eine allgemeine Frage zum Lebensstil:

14. Rauchen Sie aktuell? ❑ nein ❑ ja, seit ______ Jahren

Rauchen Sie täglich? ❑ nein, ____ (Anzahl pro Woche) ❑ ja, ____ (Anzahl pro Tag)

Wenn nein, haben Sie früher geraucht? ❑ nein ❑ ja

Wenn ja, wie viele Jahre haben Sie geraucht? (Anzahl der Jahre) ____________________

Wie viele Zigaretten pro Tag? (Anzahl Zigaretten pro Tag) ____________________

In welchem Alter haben Sie aufgehört zu rauchen? (Alter) ____________________

V Ihre persönliche Meinung ist gefragt:

15. Wenn Sie nicht regelmäßig sportlich aktiv sind, bitte nennen Sie Ihre wichtigsten drei Gründe, warum nicht:

1. ____________________

2. ____________________

3. ____________________

❑ **Kein bestimmter Grund / weiß ich nicht**

**16. Wenn Sie bisher wenig aktiv sind, was würde Sie motivieren, aktiver zu werden?
Bitte nennen Sie Ihre wichtigsten drei Gründe:**

1. ____________________

2. ____________________

3. ____________________

❑ **Kein bestimmter Grund / weiß ich nicht**

**17. Welche Sportarten wären weiterhin für Sie interessant?
Bitte nennen Sie bis zu drei 3 Sportarten, die Sie gerne machen würden:**

1. ____________________

2. ____________________

3. ____________________

Vielen Dank für Ihre Unterstützung !
